# Darkness: A Crucial Factor in Fungal Taxol Production

**DOI:** 10.3389/fmicb.2018.00353

**Published:** 2018-03-02

**Authors:** Sameh S. M. Soliman, Manish N. Raizada

**Affiliations:** ^1^Sharjah Institute for Medical Research, College of Pharmacy, University of Sharjah, Sharjah, United Arab Emirates; ^2^Faculty of Pharmacy, Zagazig University, Zagazig, Egypt; ^3^Department of Plant Agriculture, University of Guelph, Guelph, ON, Canada

**Keywords:** fungal Taxol, light, opsin, pigmentation, expression

## Abstract

Fungal Taxol acquired lots of attention in the last few decades mainly because of the hope that fungi could be manipulated more easily than yew trees to scale up the production level of this valuable anticancer drug. Several researchers have studied diverse factors to enhance fungal Taxol production. However, up to date fungal Taxol production has never been enhanced to the commercial level. We have hypothesized that optimization of fungal Taxol production may require clear understanding of the fungal habitat in its original host plant. One major feature shared by all fungal endophytes is that they are located in the internal plant tissues where darkness is prominent; hence here the effect of light on fungal Taxol production was tested. Incubation of Taxol-producing endophytic SSM001 fungus in light prior to inoculation in Taxol production culture media showed dramatic loss of Taxol accumulation, significant reduction in Taxol-containing resin bodies and reduction in the expression of genes known to be involved in Taxol biosynthesis. The loss of Taxol production was accompanied by production of dark green pigments. Pigmentation is a fungal protection mechanism which is photoreceptor mediated and induced by light. Opsin, a known photoreceptor involved in light perception and pigment production, was identified in SSM001 by genome sequencing. SSM001 opsin gene expression was induced by white light. The results from this study indicated that the endophytic fungus SSM001 required the dark habitat of its host plant for Taxol production and hence this biosynthetic pathway shows a negative response to light.

## Introduction

Taxol production has been reported from several endophytic fungi (Zhou et al., 2010); however, the mechanism and the factors affecting its production are rarely investigated. Recently, Soliman et al. (2015) demonstrated that Taxol is sequestered in resin bodies to protect plant cells from the cytotoxic effect of Taxol during transport from the site of biosynthesis to the site of storage. Furthermore, the Taxol-containing bodies are transported through the parenchyma rays to the outer tissues, the bark, in order to block pathogen invasion when plant crack openings occur (Soliman et al., 2015). On the other hand, fungal Taxol production is enhanced when cultured in conditions that mimic the internal plant habitat such as crude plant metabolite extracts and plant defensive substances (Soliman and Raizada, 2013).

Taxol-producing endophytic fungi are unstable and may lose Taxol production activity after certain generations (Soliman et al., 2011; Venugopalan and Srivastava, 2015). Several factors have been studied in order to enhance fungal Taxol production (Metz et al., 2000; Flores-Bustamante et al., 2010; Somjaipeng et al., 2016; Qiao et al., 2017). However, the major factor responsible for the loss of Taxol production activity by fungi has never been investigated. Furthermore, the fungal original habitat as a factor that can affect fungal Taxol production is rarely studied.

In general, the shared characteristic of all fungal endophytes is their prominent dark habitat in the internal host plant tissues. Furthermore, light is considered as a crucial factor in fungal metabolite production since the fungal master regulator, LaeA/VeA (Bok and Keller, 2004; Sarikaya Bayram et al., 2010; Kim et al., 2013), is mainly controlled by light (Bayram et al., 2008). Here in this study, the effect of white light on fungal Taxol production was tested. Isolation of the fungal endophyte followed by immediate light treatment significantly affected later Taxol production.

## Materials and Methods

### Materials

The following reagents were from Sigma (United States), including Taxol standard (# T7402) and fungal nutrient media: yeast-peptone-dextrose (YPD) (# Y1375) and potato-dextrose-agar (PDA) (# 70139).

### Fungal Isolation and Genotyping

Previously isolated Taxol-producing endophytic fungus (*Paraconiothyrium* SSM001 spp.) from *Taxus × media* plants cultivated on the University of Guelph Main Campus and Arboretum (Guelph, ON, Canada) was used in this study. The fungal genotyping was performed using internal transcribed spacer regions (ITS) sequence of 18S rDNA as previously described in Soliman et al. (2011).

### Effects of Light on Taxol Yield

Purified fungal tips were transferred onto fresh PDA plates at 25°C and incubated for 1 week either in full darkness or full light (white fluorescent light, 300 μmol m^-2^ s^-1^ at plate level) (Fett-Neto et al., 1995) prior to inoculation into 500 mL liquid YPD broth in 2 L flasks. In parallel, fungal hyphal tips were inoculated onto microscopic slides covered with a thin film of PDA and allowed to grow in full light for different time periods (3, 5, 6, and 7 days) prior to inoculation for fungal Taxol production and detection. Control fungal cultures fully grown in darkness were employed. Furthermore, a small hyphae tip was inoculated onto PDA plates and completely covered with aluminum foil except a small wedge was exposed to full light for 1 week at 25°C prior to inoculation into liquid YPD broth culture.

### Production, Extraction and Identification of Fungal Taxanes

Fungal tips from 1-week old pure PDA plate fungal cultures were used for production and extraction of fungal taxanes as described previously (Stierle et al., 1993; Soliman et al., 2011). Briefly, 1 mg of 1-week-old fungal tips was inoculated into 500 mL YPD broth in 2 L Erlenmeyer flasks and incubated in darkness at room temperature for 21 days. The culture was filtered and the filtrate was extracted with chloroform: methanol (9:1 v/v). The organic layer was separated, washed, and evaporated until dried. The residue was dissolved in 30 μL methanol, and 10 μl was spotted onto TLC silica gel plates (10 cm × 20 cm, Fisher Scientific #4861-320) alongside Taxol standard at a concentration of 10 μg/mL. TLC plates were then developed in chloroform/methanol (5.0:0.5) and visualized with 0.5% vanillin/sulfuric acid reagent. Fungal Taxol in the extracted liquid media was identified as previously described by Soliman et al. (2011). For Taxol quantification, 10 μL of the total extract was injected into HPLC according to Soliman et al. (2011, 2013). The peak area of each sample injected was measured by a UV detector at 233 nm and then factored against a calibration curve generated from injecting different Taxol concentrations.

### Reverse-Transcription PCR

Fungal RNA was prepared followed by cDNA synthesis as described in Soliman et al. (2015). Amplification conditions and the relative expression ratio was calculated according to Soliman and Raizada (2013). For amplification of 3-hydroxy-3-methylglutaryl-CoA synthase, the following primers were used: HMGCoASF1, 5′-ACACGAAGACTTAGCAGGTGGGTGCG-3′ and HMGCoASR1, 5′-CGAGTACCCCGTCGTCGATGGTGGTC-3′. For 3-dehydroquinate synthase ampli-fication, the primers were QuinF1, 5′-TGTAGCCTTCGCGAGGATCTCCTCG-3′ and QuinR1, 5′-ACTACACGAGCTTACTCCCGATGTGCC-3′ (Soliman et al., 2011). For opsin, the primers were OpF1, 5′-ATCAACGCAGAGTAAAGACAGTG-3′ and OpR1, 5′-TACTTTCTGCGTTGATACCA-3′. For relative normalization of gene expression, fungal 18S rRNA specific primers were used: 18SrDNA-RtF (5′-GGCATCAGTATTCAGTTGTC-3′) and 18SrDNA-RtR (5′-GTTAAGACTACGACGGTATC-3′) (Fang and Bidochka, 2006).

### Light Microscopy

Fungal hyphal tips were transferred onto microscopic slides containing a thin film of PDA media under aseptic conditions and were left to grow for 1 week prior to microscopic examination. Fungal hyphae on the microscopic slides were stained for 1 h with Sudan IV in 50% ethanol followed by light microscopy.

### Statistical Analysis

The data obtained was graphed using Graph Pad 5.0. The effects of light on Taxol and resin quantification were analyzed by one-way analysis of variance (ANOVA) using Dunnett’s Multiple Comparison Test. *P*-value < 0.05 was considered as significant.

## Results

### Light Exposure Inhibited Fungal Taxol Production

**FIGURE 1:**
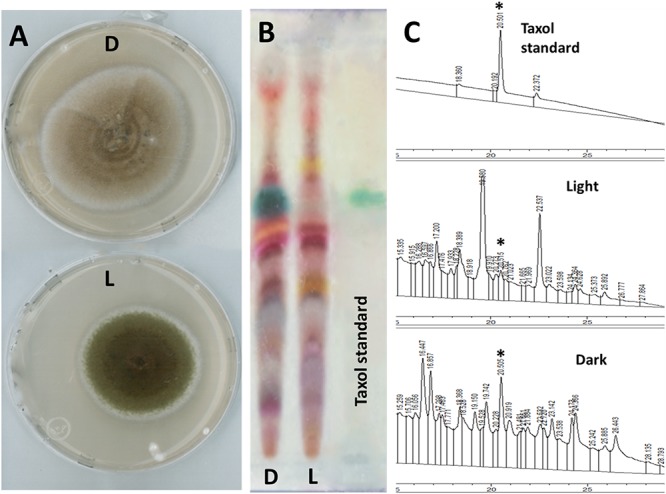
Light pre-incubation inhibited fungal Taxol production. **(A)** Growth of Taxol-producing endophyte SSM001 fungus in light (L) and darkness (D) on PDA at 25°C for 1 week. **(B)** Detection of extracted fungal Taxol after inoculation for 3 weeks in liquid YPD broth on TLC-silica plates (10 μL sample, developing system chloroform: methanol; 5:0.5 and visualized using 0.5% vanillin/sulfuric acid reagent). **(C)** Detection and quantification of fungal Taxol by HPLC-UV when fortified with 5 ng standard Taxol. The peak area of each sample (10 μL injection volume) was measured at 233 nm. The quantification data display the mean of three replicates. The asterisk is the diagnostic peak of Taxol.

Culturing purified SSM001fungus onto PDA plates under light exposure for 1 week caused production of dark green pigments covering the fungal hyphae compared to pigment-free fungus when grown in darkness (**Figure 1A**). Post-inoculation of hyphae tips from each fungal growth source into YPD broth showed production of Taxol only from the fungus previously grown in darkness (D) compared to undetectable Taxol from the culture grown in light (L). Fungal Taxol detection was performed on TLC plates (**Figure 1B**) and by HPLC (**Figure 1C**).

### Light Exposure/Green Pigmentation Is Inversely Correlated to Taxol Production

**FIGURE 2:**
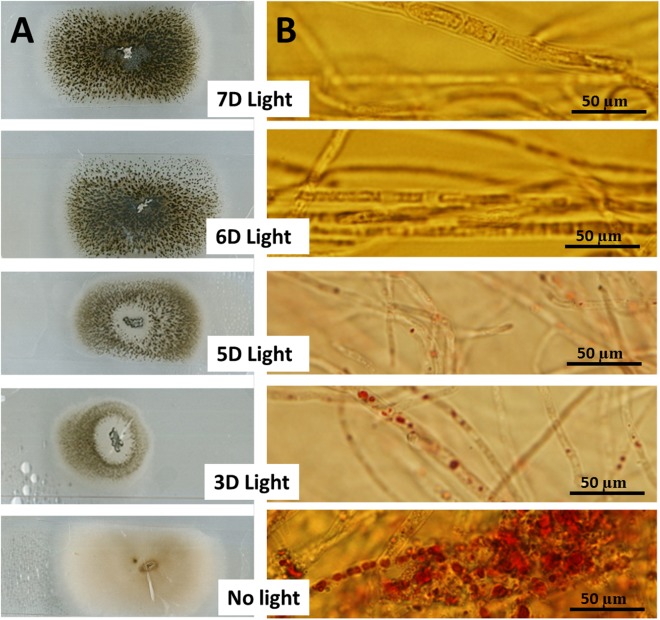
Fungus exposed to different durations of light affects resin body production. **(A)** Incubation of fungal hyphae tips on microscopic slides covered with a thin film of PDA exposed to different light durations compared to dark incubation for 1 week at 25°C. **(B)** Fungal growth from **A** stained with Sudan IV for 1 h prior to detection of resin bodies (known as sequestering organelles for fungal Taxol) visualized using a light microscope.

**FIGURE 3:**
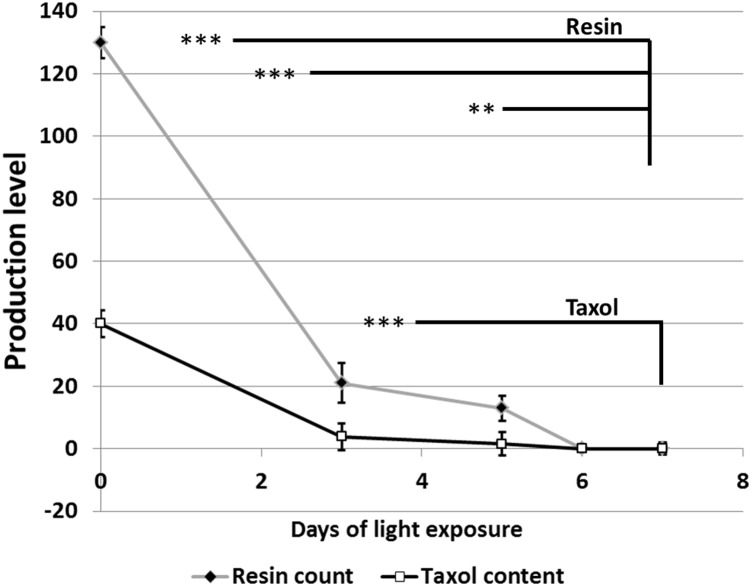
Quantification of resin bodies (absolute number) and Taxol (μg/L) in response to different time periods of light exposure. Resin body counting was performed as follows: 1 mm^2^ of fungal hyphae grown on microscopic slides coated with PDA and stained with Sudan IV was chosen from at least six different locations and counted using light microscope. Taxol quantification was performed using HPLC after inoculation of 1 mg of fungal hyphae into 500 mL PD broth. The data display the mean ± standard error of the mean. The statistical significance was calculated with one-way ANOVA and the significance level indicated by asterisks (^∗∗^*P* = 0.0005 and ^∗∗∗^*P* = 0.0001).

Growth of fungal hyphae at different light durations was accompanied by the production of different levels of dark green pigments based on the duration of light exposure (**Figure 2A**). On the other hand, duration of light exposure was inversely correlated with accumulation of resin bodies (**Figure 2B**) and fungal Taxol production (**Figure 3**). Fungal Taxol is known to be localized to resin bodies to protect plant cells from the cytotoxic effects of Taxol (Soliman et al., 2015).

### Localized Light Exposure Caused Loss of Taxol Production

**FIGURE 4:**
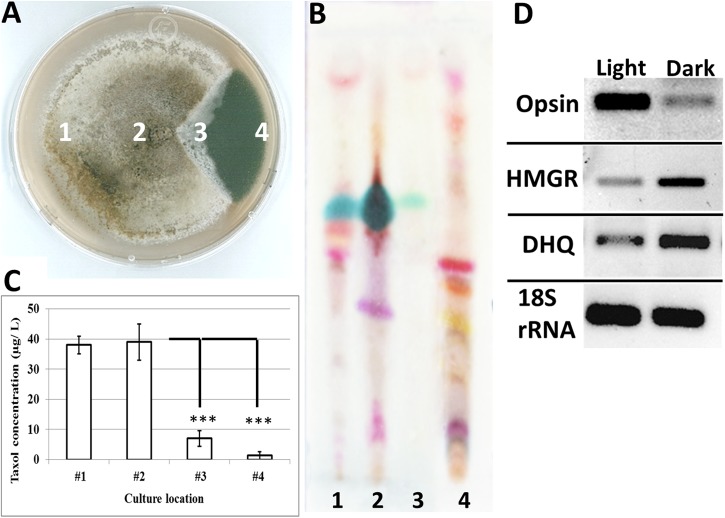
Dark-incubated versus light-incubated fungal hyphae tips. **(A)** Incubation of a fungal tip under both light and darkness caused production of dark pigments only from the place exposed to white light. **(B,C)** Detection of fungal Taxol inoculated from different locations of the same fungal hyphae on **(B)** a TLC-silica plate and by **(C)** HPLC. HPLC quantification of fungal Taxol is represented in a column graph showing the mean ± standard error of the mean. **(D)** Gene expression of opsin, HMGR and DHQ genes in both complete light (location # 4) and complete darkness (location #1). The asteriks denote a significant change in mean expression (at *P* < 0.05).

Fungal hyphae were grown in complete darkness, except for exposure of a small wedge to full light (**Figure 4A**). The light-exposed wedge was associated with production of dark green pigments (**Figure 4A**). Furthermore, Taxol was mainly produced from the hyphae located away from the light (**Figures 4B,C**). Taxol production was completely lost from hyphae tips fully grown under light (**Figures 4A–C**). Furthermore, gene expression of fungal HMGCoA reductase (HMGR) and 3-dehydroquinate synthase (DHQ), genes known to be involved in Taxol biosynthesis (Soliman et al., 2011), were significantly reduced (**Figure 4D**) in response to light exposure. On the other hand, gene expression of opsin, a gene identified from fungal genome sequencing (Soliman et al., 2011, 2015) was significantly increased under light (**Figure 4D**). Opsin is a photoreceptor gene involved in light perception and fungal pigmentation (Avalos and Estrada, 2010).

## Discussion

The results from this study demonstrate that light exposure of Taxol-producing endophytic SSM001 fungus is accompanied by the production of dark green pigments; and causes complete loss of Taxol production. Previously, it was reported that light stimulates the fungal production of specific plant-associated biomolecules in particular pigments and chlorophyll (Fang and Bidochka, 2006). Furthermore, light-grown *Taxus* suspension cultures showed significant reductions in Taxol production compared to those grown in dark (Fett-Neto et al., 1995). In another report, light inhibited nicotine production from tobacco plant tissues, and the inhibitory effect increased upon increased intensity and length of exposure (Ohta and Yatazawa, 1978).

Regarding the effect of light on fungal metabolite production and in accordance with the results of this research, light shows inhibitory effects on fungal metabolite production. The inhibitory effect is attributed to unavailability of LaeA nuclear protein (a master regulator required for fungal metabolite biosynthesis) and hence inactivation of secondary metabolite production (Kim et al., 2013).

The reason why SSM001 turns to dark green color upon exposure to light can be explained as fungal protection and defense mechanisms. Fungal melanization and pigmentation are known to provide protective mechanisms against stress factors including ultraviolet radiation (Rosa et al., 2010; Volling et al., 2011). On the other hand, many photoreceptors have been identified and characterized in fungi, including opsin (Avalos and Estrada, 2010). Photoreceptor mutation significantly reduces pigment production (Kim et al., 2014). Furthermore, light exposure reduces gibberellin metabolite production by *Fusarium* fungus, a mechanism mediated by the opsin receptor (García-Martínez et al., 2015).

**FIGURE 5:**
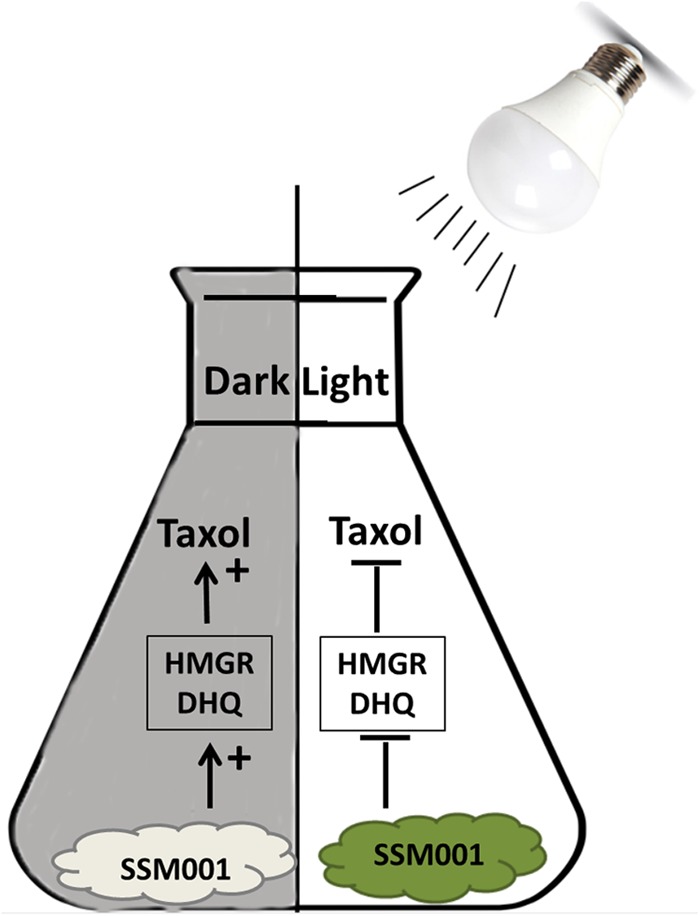
A model representing the effect of dark maintenance versus light exposure on fungal Taxol production. Maintaining the dark condition of the isolated fungus promotes Taxol production. Taxol production is mediated by increased expression of HMGR and DHQ genes. However, light pre-exposure inhibits Taxol production and is accompanied by the production of dark green pigments.

Combining the results from this research and the results from Soliman et al. (2015), it is clear that the original location of the fungus in the deep parenchyma rays of the vascular system of the plant shoot (where darkness is prominent) allow a suitable condition for Taxol production. Even though xylem and phloem in the plant vascular system might partially conduct light in both stems and roots, parenchyma cells do not (Sun et al., 2003). Furthermore, light is significantly reduced in deep plant tissues (Smith, 1995). On the other hand, it was demonstrated that plant Taxol is biosynthesized in the vascular tissue and transported through ray parenchyma to the outer tissues (Strobel et al., 1993). These observations are consistent with the results of this study, wherein continuous maintenance of fungal culture *in vitro* under conditions that simulate those of its plant host allowed for Taxol production. However, once the fungus was released outside its native dark habitat and exposed to light it lost Taxol production activity and protected itself by producing defensive pigments (model, **Figure 5**). In its natural habitat, the plant tissues provide shelter and protection for the endophytic fungus (Rodriguez et al., 2008) and hence the fungus does not require pigment production and instead can focus its metabolic resources on complementing the plant’s defence system by producing Taxol (Mariño et al., 2007) to combat fungal pathogens of its host plant (Soliman et al., 2015). By contrast outside the plant and in particular under light the endophyte protects itself by producing light-protective pigments and diverts metabolic resources away from the biosynthetic pathway for Taxol which is no longer required in the absence of a plant host. This conclusion confirms the results of Soliman et al. (2013), who showed that when conditions are provided that simulate the plant host, then fungal Taxol production is enhanced (Soliman and Raizada, 2013).

## Conclusion

Careful study of the original habitat of the endophytic fungus could be a way forward to enhance the production level of fungal-derived Taxol. Here in this study, darkness was found to be a crucial factor for fungal Taxol production. Light exposure may contribute to the instability observed in fungal Taxol production.

## Author Contributions

SS conducted the research, designed the experimental procedures, interpreted the data and wrote the manuscript. MR designed and edited the manuscript.

## Conflict of Interest Statement

The authors declare that the research was conducted in the absence of any commercial or financial relationships that could be construed as a potential conflict of interest. The reviewer PN and handling Editor declared their shared affiliation.
